# South West Orthopaedic Club

**Published:** 1984-10

**Authors:** 


### South West Orthopaedic Club

### Meeting at Plymouth, May 1984

### GARY HAMPSON MEMORIAL LECTURE THE INFLUENCE OF CONTROLLED MECHANICAL STIMULATION ON THE RATE OF FRACTURE HEALING

**A. E. Goodship, Bristol**

Despite recent advances in orthopaedic research, treatment of certain types of long bone fracture still represents a significant clinical problem. The current trend in treatment technique has been toward a less rigid fixation in an attempt to promote callus and encourage early union, using semi-rigid plates or external skeletal fixation frames with variable rigidity. It has been well documented in general terms that the pattern of fracture healing is influenced by the mechanical environment at the fracture site. However, the exact response of the differentiating callus to specific mechanical variables has not been determined.

Previous studies on stress related bone remodelling in intact bones have provided the data that form the basis for this study. Using strain gauges attached to bone surfaces in a number of species, including man, it has been shown that the normal mechanical stimulus to bone tissue is that of intermittent cyclical deformation. Both experimentally and clinically it has been established that bone tissue is dynamic and responds to changes in mechanical stimulation. An increase in the level of stimulation results in an increase in bone cell activity and deposition of bone matrix. Conversely, a decrease in stimulus produces a corresponding reduction in bone tissue. Experimental studies have shown that as few as thirty-six cycles per day of an osteogenic strain regime produce maximal new bone formation and that there is a high positive correlation between strain rate and osteogenic response.

Almost all types of fracture treatment provide some degree of artificial structural support resulting in stress protection to the fracture site, thus depriving the cells of their customary mechanical stimulus. In this study an osteogenic mechanical stimulus was applied to experimental fractures stabilised using a modified Oxford external fixator.

In two groups of sheep a 3 mm gap was created in the tibial diaphysis and controlled using the external fixator in a rigid configuration. In Group 1 sheep this rigid configuration was maintained throughout the period of healing. Fractures in Group 2 animals were treated in the same manner except that a controlled mechanical stimulus was applied to the fractures via the fixator for a short period each day. The stimulus used incorporated the following characteristics:

(1) A low force (360N) such that the fatigue stress limit of the bone/screw interface was not exceeded.
(2) A repetition frequency of 0.5 Hz.
(3) A strain rate of $30–35 \times 10^{-3}$ µe/sec.
(4) An initial axial displacement of 1 mm.
(5) A total daily stimulation of 500 cycles.

Both groups were assessed during the healing period and at post-mortem using radiographic, mechanical and histological techniques.

In Group 1 sheep callus was evident radiographically at four weeks, whereas in the stimulated group considerable callus was seen within fourteen days of operation. Mechanical assessment of fracture stiffness during healing showed a greater rate of increase in the stimulated group. This became statistically significant at 8–10 weeks post-operatively. At the end of the allotted 12 weeks healing period post-mortem mechanical tests showed a statistically significant increase in torsional strength of the stimulated fractures. Histologically the differentiation of callus appeared to be more advanced in the stimulated group, although this was only examined at one time point at the end of the 12 week healing period.

These results indicate that the prevailing mechanical environment in the early stages of fracture healing represents a very important influence upon initiation of osteogenesis and the subsequent pattern of fracture healing.

On this basis of this study a limited human clinical trial is currently in progress. It is postulated that the application of this technique may not only reduce the incidence of delayed and non-union in complicated fractures, but also enable long bone fracture healing to be initiated in bed-bound patients suffering from multiple injuries. Experimental work is continuing to evaluate the effect of each individual mechanical variable on the pattern of fracture healing.

### THE USE OF EXTERNAL FIXATION FOR COMMINUTED COLLES FRACTURE

**A. Baker, Plymouth**

This paper describes a technique of external fixation of unstable fractures of the distal radius using a dorsal quadrilateral frame and a volar plaster of Paris slab.

Details of the fixation technique are discussed with emphasis on those factors considered important in establishing a strong pin-to-bone interlock and a stable reduction of the fracture. Early post operative mobilisation is ensured by supervised physiotherapy so active and passive finger movements are established before discharge from the ward.

Good fixation was obtained in a consecutive series of eight patients. Despite the presence of osteoporosis in some cases, there was no significant loss of fracture reduction.

The clinical results are described in terms of pain, function, time off work where relevant, and range of associated joint movements.

The consistently good results obtained in this small series of patients encourage the continued use of this technique for treatment of unstable fractures of the distal radius.

### THE OUTCOME OF THE INFECTED ARTHROPLASTY OF THE KNEE

**David Johnson and Gordon Bannister, Bristol**

Infection of an arthroplasty of the knee is a major problem. Little is known of the outcome of the various treatments. Analysis of a consecutive series of 471 arthroplasties which had a minimum of 1 years follow up was performed. Hinged and non hinged prostheses with cement fixation were used with antiobiotic prophylaxis.

Twenty-three cases of superficial and 25 cases of deep infection were defined. The incidence of deep infection was greater in revision arthroplasties. Three predisposing factors to deep infection were identified; rheumatoid arthritis (p 0.05), hinged prostheses (p 0.01), and the pre-existing presence of superficial infection (p 0.001).

All patients with superficial infection obtained pain free gait within 5 months; 17 of the 25 deeply infected cases achieved pain free gait.

Deep infection was initially treated by long term antibiotics for a mean duration of 1.3 years. The symptoms of two patients settled during this period. Excision of a sinus tract was undertaken unsuccessfully 4 times. Debridement was unsuccessful in all 27 attempts, 2 split skin grafts failed. Gastrocnemius musculocutaneous flaps provided skin cover for an exposed prosthesis in 2 of 3 cases. Exchange arthroplasty failed upon two occasions. Amputation was performed 4 times. Arthrodesis was attempted 12 times, bony ankylosis was attained in 6 cases after a mean duration of 10 months immobilisation. More importantly, pain free gait was achieved in 11 of the 12 cases.

In conclusion, deep infection in a knee arthroplasty is eradicated by long term antibiotics alone in only 8% of cases. Whereas arthrodesis provided the pain free gait that these unfortunate patients desire in 92%.

### SELECTIVE FRACTURE STRESS USING EXTERNAL FIXATION

**P. May, Bath**

Simple, stable, transverse fractures and grossly comminuted, unstable fractures impose requirements for their early stabilisation that need separate external fixator technique.

The biomechanical investigations of Kempson, Burny but especially Chao have established stiffness characteristics of many external fixator parameters. With the Vidal-Hoffman frame Chao derived a comparative stiffness-factor (K) composed of bending, torsional and compression elements.

But do we want an overall stiff frame? Strength in compression is created by design features which then resist extrinsic compressive stress ... the latter is physiological and may be *useful* in treating comminuted fractures. So a frame suitable for compressing a stable fracture may not be the best for a comminuted one that cannot be mechanically compressed in its early stages.

A mechanical investigation is presented using a laboratory model tibia with standard fracture supported by standardised external fixator systems. The only variable was the pin angle in the plane of the bone's cross sections. Not only are the classic single and double sided devices compared but intermediate designs using two single bars at various angles to each other.

Double sided frames are always weaker to perpendicular (saggital) bending stress than to pin-plane (coronal) stress. Bending stiffness in all planes can be equalised by the inovation of a 'V-90' device ... two half-pin bars, one saggital, one coronal.

Extrinsic compression is shown to affect fractures most through single bar and angulated double bar designs but is resisted most of the common, transfixing pin, double bar device.

The relationship of external physiological stress to fracture strain with different designs is shown. Theoretically we can now adjust, independently, design features controlling the four modes of fracture strain.

Application of this aspect of external fixator theory will be discussed in the light of present knowledge about which types of movement (or strain) are beneficial to fracture healing.

### DISLOCATIONS OF THE CARPAL LUNATE

**Ian D. Rawlings, Plymouth**

This paper consists of a review of the results of treatment of some 30 patients, who suffered an anterior dislocation of the lunate, without associated carpal injury. It therefore does not include trans-scaphoid peri-lunate dislocations.

The result of this review, showed that only 43% of the cases achieved an acceptable outcome.

Two main factors were identified, influencing the results significantly and these were:

1. Delay in diagnosis.
2. Adequate reduction.

Suggestions are made as to methods of ensuring earlier diagnosis and also means of assessing the adequacy of reduction. It is anticipated, that adherence to these suggestions will greatly improve the number of satisfactory results obtained in the treatment of this injury. A prospective study is in progress to evaluate these suggestions.

### IN-VITRO STUDY FOR CEMENTING TECHNIQUES OF THE FEMORAL COMPONENT IN TOTAL HIP REPLACEMENT

**M. Halawa, Plymouth**

Previous in-vitro studies done with the team at Exeter University, had shown that low viscosity cement introduced under pressure increases the load at failure of the cement bone junction in cylindrical femoral specimens. Different techniques for filling the femur with low viscosity cement have been practised. With the aim of creating an increase in pressure at the cement bone interface during injection of the cement. Paired femora were prepared using different techniques for filling the femur with two different brands of cement, one of moderate viscosity (Simplex B), the other of high viscosity (C.M.W.). Four pressure transducers were inserted at the cement bone interface to give pressure recordings while filling the femur and while inserting femoral component of the Exeter hip.

Push out tests were done following this technique to show the load at failure relative to each technique.

This preliminary strudy has shown that the higher the viscosity, the higher the pressure at the interface between cement and bone and the higher the cement bone load at failure.

The main reason that low viscosity cement does not produce high pressure at the interface is the lack of satisfactory techniques to seal the top of the femur during cement filling and during prosthetic stem insertion.

